# Attitudes and perceptions about breastfeeding among female and male informal workers in India and South Africa

**DOI:** 10.1186/s12889-020-09013-9

**Published:** 2020-06-05

**Authors:** C. Horwood, A. Surie, L. Haskins, S. Luthuli, R. Hinton, A. Chowdhury, N. Rollins

**Affiliations:** 1 Centre for Rural Health, University of KwaZulu-Natal, Durban, South Africa; 2 Indian Institute for Human Settlements, New Delhi, India; 3 RHEdit, Geneva, Switzerland; 4 Department of Maternal, Newborn, Child and Adolescent Health, World Health Organization, Geneva, Switzerland

**Keywords:** Breastfeeding, Working women, Workplace health, Maternal health, Child health, South Africa, India, Informal economy

## Abstract

**Background:**

Worldwide, over 740 million women make their living in the informal economy and therefore lack formal employment benefits, such as maternity leave, that can improve infant feeding practices. Returning to work is one of the biggest challenges women face to maintaining breastfeeding. This study aimed to explore attitudes and perceptions towards breastfeeding in the informal work environment among male and female informal workers.

**Methods:**

The study used a qualitative research design. Purposive and snowball sampling was employed. Focus group discussions (FGDs) were conducted among men and women working in different types of informal jobs, in India and South Africa. Data was analysed using a thematic approach and the framework method.

**Results:**

Between March and July 2017, 14 FGDs were conducted in South Africa and nine in India. Most women were knowledgeable about the benefits of breastfeeding and reported initiating breastfeeding. However, pressures of family responsibilities and household financial obligations frequently forced mothers to return to work soon after childbirth.

Upon return to work many mothers changed their infant feeding practices, adding breastmilk substitutes like formula milk, buffalo milk, and non-nutritive fluids like Rooibos tea. Some mothers expressed breastmilk to feed the infant while working but many mothers raised concerns about expressed breastmilk becoming ‘spoilt’. Breastfeeding in the workplace was challenging as the work environment was described as unsafe and unhygienic for breastfeeding. Mothers also described being unable to complete work tasks while caring for an infant. In contrast, the flexibility of informal work allowed some mothers to successfully balance competing priorities of childcare and work.

Sociocultural challenges influenced breastfeeding practices. For example, men in both countries expressed mixed views about breastfeeding. Breastfeeding was perceived as beneficial for both mother and child, however it was culturally unacceptable for women to breastfeed in public. This affected working mothers’ ability to breastfeed outside the home and contributed to a lack of respect for women who chose to breastfeed in the workplace.

**Conclusion:**

Mothers working in the informal sector face multiple challenges to maintaining breastfeeding. Interventions are required to support feeding and childcare if global nutrition and development goals are to be met.

## Background

The Global Strategy for Women’s, Children’s and Adolescents’ Health (2016–2030) promotes a vision of health and development centred around the three objectives of Survive, Thrive and Transform [1]. Improving protection, promotion, and support of breastfeeding contributes to the realization of both the Global Strategy and the 2030 sustainable development agenda, as optimal breastfeeding is critical for reducing morbidity and mortality as well as for enhancing child development, intelligence and human capital, including school attainment [2, 3]. Mothers also benefit. Breastfeeding decreases the risk of mothers developing breast cancer, type 2 diabetes and heart disease, and aids in birth spacing [2]. Optimal breastfeeding, as defined by WHO, includes exclusive breastfeeding to 6 months followed by continued breastfeeding to 2 years and beyond, with the introduction of nutritious complementary feeds from 6 months of age [4]. Although the benefits of breastfeeding are well-documented, globally only 41% of infants less than 6 months of age are exclusively breastfed [5].

The determinants of breastfeeding practices are multifaceted and operate at social, structural and individual levels [3]. It was been widely shown that among women working in the formal sector returning to work is one of the biggest challenges women face to maintaining exclusive breastfeeding [6, 7]. A variety of legislative and policy measures can improve breastfeeding among working mothers, including paid maternity leave and breastfeeding-friendly workplaces. However, worldwide close to 60% of working  women make their living in the informal economy which is not regulated or protected by the state [8], with the result that this population of mothers do not benefit from formal employment-related protections that are known to improve infant and young child feeding practices [9]. A survey conducted in South Africa suggests that despite good knowledge of the importance of breastfeeding and high rates of breastfeeding initiation, many informal workers stop breastfeeding when returning to work [10].

What’s more the informal economy is continuing to grow in most regions and in urban settings. Over 90% of the world’s informal employment is in emerging and developing countries [8]. While definitions of “informal sector,” “informal work” and the “informal economy” vary considerably, informal workers are often described as those who do not have any job security, income security or social security and are therefore extremely vulnerable to exogenous shocks [11]. The informal economy is heterogenous in its numerous sectors, variety of employment statuses and places of work. It includes contributing family workers and self-employed own account workers such as street vendors, waste pickers, construction workers, home-based workers and employed domestic workers [9, 12]. Informal workers struggle with poor housing, lack of basic services such as electricity and water, and extreme overcrowding. They are particularly neglected when it comes to health [13].

Women are overrepresented in self-employed informal occupations with a lower chance of high returns and worse working conditions [8]. In sub-Saharan Africa, Latin America and Southern Asia, informal employment is a greater source of employment for women than for men [8]. Women workers in the informal economy face a higher risk of poverty than those in the formal economy and do not have the same degree of access to health care [8]. Women generally take the greater responsibility for childcare in the household, and this may reduce women informal workers’ earning ability, forcing them to seek flexible work that is less well paid [14]. While there is some evidence of an association between job informality in the Global South and poor health among women workers [15], we still know very little about how informal work affects maternal, newborn and child health. There is compelling evidence that growing up in poverty has a negative impact on child outcomes, including health, development and long term achievement [16].

This study explores the attitudes and perceptions towards breastfeeding in the informal work environment among male and female informal workers in South Africa and India. It examines the experiences of women informal workers of breastfeeding and caring for children while continuing to work and the challenges they face. It is one part of a mixed methods study and multi-country collaboration to better understand breastfeeding and childcare practices among women informal workers, and to develop appropriate strategies in response [17]. Increasing the proportion of infants exclusively breastfeed is a major target of the 2016–2025 Decade of Action on Nutrition [18], and we must address the needs of this somewhat invisible and growing population if global nutrition goals are to be met. However, to our knowledge there is no published data describing experiences of breastfeeding among informal workers.

## Methods

The study used a qualitative design as part of a broader mixed-methods study [10]. The qualitative design was informed by the framework method for thematic analysis [19] which enabled the researchers to explore themes related to the commonalities of participants’ experiences and the understandings, challenges and complexities of infant feeding practices and child care within the context of informal work [20]. The qualitative design also created space for in-depth and shared dialogue between the researchers and participants [21]. We conducted the study from February to July 2017 in one urban and one rural site in KwaZulu-Natal, South Africa and in four sites in New Delhi, India. The sites were selected to reflect the heterogeneity of the informal economy, in terms of sectors and places of work represented.

### Study setting

In India, the informal economy is the dominant form of work. It makes up to 90% of the total workforce and 90% of women workers. This includes domestic workers, market traders and home-based workers. By comparison, South Africa’s informal economy makes up a third (34%) of the total workforce with a slightly higher percentage of working women engaged in informal work (35.9%) compared to men (32.5%) [8]. The informal economy is smaller than other developing countries, but it constitutes a significant source of employment in the country. Almost all domestic workers are women, accounting for around 20% of all women informal workers [22]. Workers in the informal economy in South Africa earn on average around two-thirds less than formal workers (R1 733(US$120) per month vs R5 000 (US$ 340) per month). Women make only around 75% of men’s earnings [23]. In India, informal workers can receive average daily earnings of between INR 205 (US$3) and INR 411 (US$6), compared with INR 750 (US$11) earnt by regular formal workers. As in South Africa, the labour market in India is characterized by gender-based disparities [24].

Rates of exclusive breastfeeding are low in both South Africa and India, estimated at 31.6 and 54.9% respectively [25–27]. While informal women workers in both countries lack formal job benefits, there are some state provisions in South Africa and India for new mothers. In South Africa most births occur in public health facilities at no cost, and new mothers can also apply for a child support grant of R400 per month (US$ 34) [28]. In India, women have access to monetary rewards if they opt for institutional births. They can receive up to INR 6000 (US$100) for hospital costs, immunization and nutrition through programmes like the Jananai Suraksha Yojna [29]. In addition, nutritional support for pregnant, and lactating mothers can be accessed through the Integrated Child Development Scheme [30].

In South Africa, the urban study site was Warwick Junction, an informally structured public market in central Durban. It is situated in a large transport interchange for approximately 460,000 commuters each day and between 6000 and 8000 street vendors spread across nine different market areas. The rural site was Uthukela District, one of the 11 districts of KwaZulu-Natal province. There are a range of covered markets and street markets in three small towns in Uthukela. Each market has representation from both peri-urban and rural informal workers. There is no data available about the informally working population in the district.

In India, there was an overlap between the sector of work and site of study. Informal work and informal housing are often connected in Indian cities because the costs of transport are high and informal workers look to reside close to their worksites. The study was conducted in four settlements in New Delhi and covered four sectors of work: Anand Vihar where domestic work was the dominant form of work for women; Jahangirpuri settlement for its street vending of fruit and vegetables; Raghubir Nagar settlement where women engaged in traditional *pheri* work and bartered utensils for second-hand clothes in private households and then sold the clothes in public markets; and Sundarnagri settlement for its predominantly home-based work.

### Data collection

Focus group discussions (FGDs) were used to collect data. This methodology allowed for discussions on a specific topic of interest with a relatively small number of participants from a similar sociocultural background [21]. A FGD guide was developed to explore infant feeding practices of mothers working in the informal economy and the perceived role of women within the informal economy (supplementary file 1). The guide also focused on the care of children in the workplace or while mothers are at work, and attitudes to breastfeeding in the informal work environment. Different FGD guides were used for women and for men (supplementary file 2), and the guides were adapted as required for each setting. We conducted FGDs with women and with men in different types of informal work, including domestic workers, street traders and waste pickers, in order to obtain different perspectives about breastfeeding from different types of informal workers. A recent study in Durban suggested that different categories of informal workers face different challenges in feeding and caring for their children [10]. Male participants were included to provide a gendered perspective about breastfeeding in the informal workplace.

In each country FGDs were conducted by experienced researchers trained in qualitative research to masters level (SL, AS, AC). All researchers were female, and currently employed as researchers by their affiliated institutions. The research teams had a complementary mix of valuable experience with the topic. The team in India had strong knowledge and experience with female workers in the informal economy and had worked closely with Self-employed Womens’ Association (SEWA) on different occasions. The research team in SA had a proven expertise in maternal and child health.

FGDs were convened according to work type among women, with separate FGDs conducted with different types of informal workers and in different areas as shown in Table 1. Each FGD included only workers with the same occupation working in the same area, and discussions with men were conducted separately. The FGDs were held in central venues away from the workplace, such as local halls, municipal offices or small hired venues. Discussions took place in participants’ language of preference, either English or isiZulu in South Africa, or Hindi and Bengali in India. Researchers had minimal contact with participants prior to the FGDs. Participants were provided with information about the objectives of the study and the researchers role in conducting the FGDs. Only participants and researchers were present during the discussions. All FGDs were audio recorded. FGDs were continued until researchers agreed that data saturation had been reached.

**Table 1:** Description of focus group participants in India and South Africa

Type of work	Country	Setting	No. of FGDs	Gender	No. of participants
Domestic workers	India	Anand Vihar, New Delhi (ND)	2	Female	11
				Male^a^	12
	South Africa	Urban, KwaZulu-Natal (KZN)	2	Female	7
				Female	3
	South Africa	Rural, KZN	2	Female	5
				Female	9
Home-based workers	India	Sundarnagri, ND	2	Female	12
				Male^a^	11
Market traders / street vending	South Africa	Urban, KZN	3	Female	8
				Female	7
				Male	8
	South Africa	Rural, KZN	4	Female	6
				Female	4
				Male	6
				Male	3
	India	Jahangirpuri, ND	3	Female	10
				Female	12
				Male	15^a^
Waste pickers	South Africa	Urban, KZN	3	Female	8
				Female	5
				Male	8
*Pheri* bartering	India	Raghubir Nagar, ND	1	Female	9

^a^ Male participants were related to informal women workers but did not undertake the work themselves

### Participant recruitment

Women and men were eligible to participate if they were 18 years or older and had been working in the informal economy for more than 6 months. Women participants had to have a child under the age of 5 years. The research teams used purposive and snowball sampling techniques to facilitate a diverse representation of women working in the informal economy [31]. The snowball technique has been shown to be useful in engaging hard to reach populations [32]. The sampling approach aimed to recruit participants from a variety of informal jobs to include diverse viewpoints and experiences among participants.

At both sites recruitment was facilitated by local organisations working with informal workers who introduced participants to researchers. In India, SEWA supported the identification of women workers in the sectors of street vending, home-based work, traditional *pheri* bartering, and domestic work. In Durban AeT led the process of engagement to informal workers at the Warwick Junction site, participants included waste pickers, street vendors and market traders. In the Uthukela district members of the study team initiated contact with women working in the informal economy. They visited places where groups of women commonly meet, such as churches, to identify eligible participants away from the workplace and approached women in person. This included women working as informal traders as well as domestic workers. All women were invited to suggest other women from their networks or community who might be interested in participating, as well as male family members working in the informal economy.

### Ethical considerations

The research teams provided information to all participants on the purpose of the study before discussions took place. Participants were also assured of confidentiality and that they could withdraw from the study at any time. Transcripts were de-identified and included no personal information. Participants were compensated for the time spent away from the workplace. Exact payments in the different sites were agreed with local stakeholders to ensure that the amount was commensurate with lost earnings to avoid being an undue incentive. To mitigate potential risks and sensitivities among the informal workers, research teams worked with organisations in each setting that had long-standing engagement and experience of the respective communities. Feedback on study results was provided to the organisations after completion of the study.

### Data analysis

Trained members of the research teams transcribed the FGD recordings verbatim and translated the transcripts into English. The data was analysed using thematic approach and the framework method. Framework analysis was selected as a suitable approach to manage and organise the data across the two sites and to provide a systematic analysis structure which enabled commonalities and differences in the data to be identified as well as relationships between different parts of the data. This approach enabled descriptive and explanatory conclusions to be drawn around themes [19].

The research teams read and reread all participants’ accounts for a priori themes in the FGD guide as well as for themes emerging from the data. Based on this, two members of the research team in each country independently applied codes to a sample of transcripts to describe the content, key ideas and themes, such as reasons for returning to work or factors influencing breastfeeding practices. Initial coding was shared among team members via email. The research teams held meetings via Skype to further discuss the codes and the emerging patterns and themes. This process informed the development of an initial analytical framework which was applied to the remaining transcripts using the existing codes. Any new themes were discussed, and the analytical framework was further refined until no new themes were identified. A matrix was developed for each country, with data from each transcript summarized by theme. To ensure consistency, the team members compared their summary styles in the early stages of the analysis process. The research teams jointly discussed the initial findings to ensure accurate interpretation of the data. The software programme NVivo was used to manage the data analysis process (NVIVO v12, 2019).

## Results

In South Africa 14 FGDs were conducted and nine FGDs were conducted in India (see Table 1). The duration of the discussions varied between 50 min and 1 h 20 min. It was not possible to determine whether any participants refused because recruitment was done by local informal worker organisations. Demographic characteristics, including type of work, of South African and Indian participants are provided in Table 2.

**Table 2:** Participants’ demographic details

Participant demographics (***n*** = 179)	South Africa (***n*** = 87)	India (***n*** = 92)
**Population**		
African	87	–
Hindu	–	80
Muslim	–	11
Other	–	1
**Sex**		
Female	62	54
Male	25	38
**No. of days worked per week**		
1–3 days	15	9
4–6 days	47	52
7 days	25	31
**Level of education**		
Did not attend	1	42
Primary school education	15	31
Secondary education	51	14
Matriculated	20	5
**Age category**		
20–25	12	50
26–30	20	33
31–35	22	7
36–45	25	–
46>	8	–
Don’t know	0	2
**No of children under 5**		
None (males only)	12	–
1 child	62	54
2+ children	13	38
**No of children under 5 currently residing with fathers**		
Father not staying with child	26	4
Father staying with child	61	88

### Good understanding of the benefits of breastfeeding

Women in both countries strongly valued and acknowledged the importance of breastfeeding for child health and development. Most of the women reported having initiated breastfeeding after birth and showed themselves to be knowledgeable about the benefits of breastfeeding. The reported benefits for children ranged from cognitive and physical development to protection from infection or disease.

*‘What made me to choose breastfeeding is that I wanted her to grow up well, be intelligent and also have strong bones for when she starts to crawl. I wanted her to be fit and proper. A mother’s milk is important. I also could not afford to give her formula. There was no money’. Female street trader, rural, South Africa*

Mothers showed themselves to be knowledgeable about the hazards of formula feeding. Several women gave examples of how they perceived that their children were healthy because of being breastfed, or that their children became sick or did not grow well once breastfeeding was stopped.

*“Just to emphasize, breastmilk is healthy for the baby. He needs it in every way such that I am a living witness of that. My first child is now 13 years old and he’s never been to a doctor; he’s never been sick to need a doctor.” Female waste-picker, urban, South Africa*

Breastfeeding was also seen as a convenience especially in financial terms given the expense of purchasing alternatives. Mothers could also avoid the special preparation required with formula milk or other milk such as buffalo milk which had to be boiled and mixed with water. For example:

*“There is formula milk that is available, and people ask us to give the child that, but it is so expensive that we are not able to give it. Breastmilk is free. Sometimes we give the milk we get outside for Rs.10-15 to the child, but if we do not have money to buy milk, we mix sugar and water and give it to the child”. Female street/market trader, India.*

### Returning to work changed breastfeeding practices

Despite the well-known benefits of breastfeeding, on returning to work many mothers changed their infant feeding practices, including adding breastmilk substitutes. Some mothers continued to breastfeed albeit not exclusively. Others shortened the duration of any breastfeeding or ceased breastfeeding altogether. In some cases, this led to children receiving inadequate feeds and several mothers reported that they were unable to afford formula milk. They instead gave their children tea in South Africa and buffalo’s milk mixed with water in India. Several women also gave their child expressed breastmilk as an alternative to continuing to breastfeed.

*“When I went to work, I expressed for him, because I had milk I wouldn’t buy the tin (formula milk). I would express for him so that I could leave him with my milk that would cover him for the whole day until I came back” Domestic worker, rural, South Africa.*

Mothers in both India and South Africa, however, raised hygiene concerns with expressed milk. For example, women in India perceived expressed milk as “spoilt” once it had left their breast. This was coupled with the lack of infrastructure to support it, such as a fridge at home.

*“It is not a question of me feeling okay with pumping or expressing milk – the milk can go bad. When the child drinks directly from the mother that is the right way. If it (breastmilk) is exposed … then it goes bad … It becomes poison outside. Yes, there are more of disadvantages. The milk breaks. It becomes yellow and green. I have never done it, neither have I heard about anyone doing it*.” –*Domestic worker, India.*

In addition, given the poor sanitation in low-income settlements where many women lived, they were concerned the expressed milk might become contaminated.

*“They taught us at the clinic that we can express milk for the baby, but the problem is that you don’t know how the nanny will handle baby’s milk. Maybe she will get it dirty; maybe she leave it to rot all those things. So to avoid that it’s better to tell yourself that ok … … that was the reason I stopped.” Domestic worker, urban, South Africa.*

Women, particularly in the India groups, also conveyed the perception that breastfeeding stifled the independence of both mother and child. As such, many women introduced other food and mixed fed their children at an early age due to fears that child would become dependent on breastfeeding and ‘refuse anything else, even food’. *Pheri* vendors in India viewed the stopping of exclusive breastfeeding as a strategy of care so their infants could get used to their absence on return to work. For example:

*“Many mothers-in-law tell their daughters-in-law to get the infant used to buffalo (milk) in a week of delivery, so that she can resume her work again. Usually within 40 days the infant has started drinking multiple things.” Pheri vendor, India*

Similar sentiments were expressed in relation to the health and independence of the mother. Mothers noted that continued or prolonged breastfeeding impacted on their own strength and energy as well as their ability to return to work and earn for their families. To address this concern, mothers discussed different strategies for weaning their children off breastmilk. For example:

*“Some children drink breastmilk for a long time, then the mother is troubled. If the child drinks mother’s milk for a longer time, the mother becomes thin and weak and the child becomes healthy/fat. If the child is weaned after a year, then it is better. It takes a toll on the mother. My child drank up to his 3rd year. I had to apply neem [bitter] leaves on my breasts to make him stop being so dependent on my milk.” Domestic worker, India*

### Socioeconomic factors affected the timing of mothers’ return to work

Several socioeconomic factors contributed to the inability of working mothers to continue breastfeeding and delay their return to work after childbirth. This included the pressure of financial and family responsibilities, absence of a dual income in the household and lack of access to government cash grants or benefits. A woman’s ability to decide when to return to work was also determined by whether she could draw on her kinship network to provide support as a substitute for her earnings.

*“What made me go out to work is because I was tired of asking for everything from my mother. My mother works hard at the (taxi) rank. The child’s father disappeared a long time ago. He is absent. So I decided that it was not right for me to ask for everything from my mother whereas she is also supporting my two children. So I decided to go out to look for a job. It is quite difficult to be a woman. I do not want to lie.” Female street trader, rural South Africa.*

In both countries’ household income was dependant on women’s work. Women’s earnings contributed centrally to household expenses and this was a specific determinant to returning to work soon after childbirth. Many women in India indicated that their husbands were either unemployed or had irregular work and were underemployed. This forced some women to return to work as soon as 2 weeks after childbirth.

*“We take the responsibility to earn for our children’s sake. If the children need anything for their studies and my husband’s income isn’t enough, then I should be able to get it for him/her. If I would ask him to give me money for anything out of ordinary, he would get angry. I started working to earn some money to be able to cover such expenses, but soon I was taking care of most expenses.” Female market/street trader, India*

Likewise, in South Africa many women received limited or no financial support from the father of their children. In some cases, a small child support grant, eased participants’ return to work by providing enough money to sustain the household.

*“My mother helped me at home. The social grant also helped. My sister who is in Johannesburg - she is my cousin – when I called her and told her that we were starving at home and it was going to be a while before the social grant pay date, she would ask me how much I needed, and I would tell her any amount that she can afford.” Female street trader, rural, South Africa*

The benefits of informality meant women were able to take on work that was more flexible. For example, informal work afforded some pregnant women and mothers flexibility in terms of adapting their work to their circumstances. Some participants were able to change from market or street vending to home-based work (such as doing laundry) which enabled more time for rest and child care. Informal work also gave women independence and the opportunity to provide for themselves and their babies.

*“I got my last born child, when I got him I was selling brooms in Johannesburg but because I am now pregnant I wasn’t able to go to the hill and fetch the material, I stayed at home and I did laundry for people until I gave birth. I waited for 3 months, when he turned 4 months I started again to help people with their laundry and I stopped now that I’m selling mealies”. Female street trader, rural, South Africa.*

However, informal work was insecure, irregular and less well paid. It was also the only income-earning opportunity available to many women. They therefore had to also consider how taking time off work after the baby was born might impact on their work options. For example, with no protected maternity leave, or other labour protections such as trading licenses, safeguarded trading spaces, or employment contracts, there was no guarantee a woman could return to work after having a child. Working mothers were often faced with having to forgo work at a site (vending, domestic work, *pheri*, waste pickers), or with a contractor (home-based work) to find other work options. This was a significant concern for domestic workers in South Africa who often lost their jobs while on maternity leave and had to look for new service work.

*“They told me that when I’m ready I will still have my job, I should just go home and raise the baby. I raised my baby when I called them to tell them that I’m ready (to return to work) they told me to wait a bit it’s still quiet at work but there were people working … there was someone who was working on my account, but I couldn’t come back because I had a baby.” Domestic worker, urban, South Africa.*

Women in India also had concerns about loss of work after childbirth, however, their concerns were related to the limited work options available to them that were more suitable to their situation. For example, women claimed it was simple to resume self-employed work, such as street vending, but it was not always the best option for child care.

*“If I could get a home-based part time job I would be able to look after my child better. In our community women only work as pher-waalii, there is no other option. I would want to try options other than pheri.” Pheri vendor, India*

### Mothers experienced barriers to breastfeeding in the workplace

#### Sociocultural barriers

Male participants in both countries displayed an ambivalence towards women breastfeeding in the workplace. They acknowledged the value of breastfeeding and were sympathetic to women working who were breastfeeding. However, there was frequently a strong feeling among male participants that women had to cover themselves while doing so.

*“My opinion about breastfeeding by women in the market is tolerance even when it is difficult. If your neighbour is a woman, you cannot say to her “go and breastfeed somewhere else far from my table”. You have to be tolerant and she must cover up whilst the baby is feeding.” Male street vendor, urban, South Africa*

Male vendors in India spoke about the concerns of privacy and respect for women who chose to breastfeed in public spaces.

*“There are men who would whistle or tease women, but as far as I can speak for myself and the men around me there is a different opinion for breastfeeding mothers. If there is a breastfeeding mother, then most people turn away when they see what she is doing. If a woman were wearing the same clothes and standing by herself I’m sure we would look her up and down and keep staring but not for a breastfeeding mother.” Male market trader, India*

In both countries cultural attitudes towards women’s bodies contributed to a woman’s decision to cease exclusive breastfeeding or to reduce the duration of breastfeeding. In India home-based workers were able to breastfeed their children during work hours in the privacy of their homes because their home and worksite overlapped. Domestic workers strategized to breastfeed their children in the early morning, afternoon and late evening in their own homes by making frequent visits back and forth between worksite and home in a day. *Pheri* women who took their children to the market where they sold second-hand clothes faced criticism about breastfeeding in public but continued nonetheless. This was true for most of the women in India who, despite feeling embarrassed to breastfeed in public spaces, attempted to overcome their discomfort by placing a scarf or the open end of a sari across their chest when breastfeeding.

*“See, the thing is you have to cover yourself with your aanchal (part of a sari garment). What can you do? You have to feed your child. There’s no option when it comes to that.” Female Fruit and Vegetable Vendor, India.*

Women in South Africa expressed it was culturally unacceptable for women to breastfeed in public. This was reinforced by male participants who explained that cultural norms prohibit the showing of breasts in public, although young girls are permitted to show bare breasts. For example, according to Zulu culture in South Africa, women who had recently given birth or were breastfeeding were considered unclean and should not handle or serve food. It was said people felt “disgusted” by a food vendor who was a breastfeeding mother and male customers in particular would experience misfortune if they consumed her food. Many women street traders recalled having to hide when breastfeeding their children in the workplace, or otherwise “return home with all my goods not sold.”

*“Eh … let me expand by touching on culture although some of us no longer take it into consideration when working with women who recently gave birth. Working with her and buying food prepared by her is culturally unacceptable because she is supposed to be home until at least a child is 5 or six months old. … You wouldn’t come near or touch your child until about six months. Some of us still observe that ritual because if we don’t observe it, accidents befall you, even bad luck. It also weakens the muthi (medicine to strengthen manhood)” Male street trader, urban, South Africa.*

#### Structural barriers

Despite the varied work environments of participants, most work settings were perceived by male and female participants to be unsafe and unhygienic for breastfeeding and childcare. Waste pickers, in particular, were not always able to keep clean or wash themselves before breastfeeding. In India, street vendors were afraid of exposing their infants to extreme weather conditions during vending hours. The difficulties of focusing on work while caring for small children meant children could be less safe in the workplace. Participants voiced their fears about children getting lost, kidnapped, left unattended and exposed to violence and crime. Other situations included children being exposed to chemicals, faecal matter and eating out of dustbins. Several participants gave examples of where children had been injured or even killed.

*“You can’t take your child to the market. Forget the heat and the winter, someone will pick my child up! Or he will destroy all the piles of vegetables I’ve put up!” Female Fruit and Vegetable Vendor, India*

#### Logistical barriers

Some women reported that breastfeeding was time consuming and impractical to continue on return to work. Workplace conditions (a pavement for street vendors; or a private home for domestic workers) often hindered the proximity between mother and infant needed for breastfeeding. The logistics of keeping a child close were a challenge, especially for mothers who worked as waste pickers or vendors as they often walked long distances and carried heavy loads.

*“I do not take my child with me to work; I wouldn’t be able to because you sometimes have to carry a lots of cardboard; sometimes I walk a long distance say from here to that Cambridge so I am unable bring him with me.” Female waste-picker, urban, South Africa.*

Women also highlighted the practical difficulties involved with taking time to breastfeed and care for their child while trying to meet their multiple work tasks, whether it be serving customers, cleaning houses or picking waste. As a result, to avoid disruptions and the potential safety concerns described above, many women opted not to take their children to work.

*“Should I work or should I take care of my child? They’ll create a mess in the kothi and I’ll get yelled at. Even if I didn’t get yelled at, why would I want to clean double the mess in someone else’s house?” Domestic Worker, India*

## Discussion

Despite the extent of women working informally in the Global South, little is known about childcare, particularly breastfeeding practices, among women informal workers. This formative study sought to better understand breastfeeding and childcare practices among women informal workers in different settings in India and South Africa, with the intention to inform the development of strategies to improve maternal and child health and provide better income security for these women. Our findings show that, although women working in the informal sector understand the importance of breastfeeding, they are frequently unable to practice exclusive and sustained breastfeeding because of multiple adverse factors. They must return to work early to fulfil their financial obligations to the household and the family but are often unable to take the baby to work with them because of a hazardous work environment, their inability to undertake work tasks while looking after the baby, and lack of support from male colleagues. While breastfeeding was the focus for this study, we saw breastfeeding as a proxy for child health, and based on our findings we discuss possible pathways for intervention.

In our study, women informal workers did not have access to formal protections which safeguarded their time away from employment allowing them to recover from childbirth, breastfeed, be close to their infants and plan for a new life. Returning to work placed an enormous physical and emotional stress on some mothers who found it difficult to balance the need to earn an income as well as care for themselves and their child. Women were forced to decide between returning to work and devolving care of their infant to someone else or staying at home to care for the child and forfeiting income. However, it is important to note that informal work offered some of the women in this study independence and flexibility. A review of poor working women and child care provision in Africa, Latin America and Asia also suggests that poorer women may use the flexibility of informal economy work to navigate the trade-off between impoverished households, child care and earning work when their children are young. Although this allows them time to care for their children, the work is also insecure and poorly paid [15]. Women informal workers need more options for securing their incomes to delay time to return to work. Possible approaches include saving schemes, safeguarding trading spaces, and community support for childcare to free women’s time to increase their productivity.

The stress of balancing this ‘double-burden’ of earning an income and shouldering the main responsibility for childcare was significant for women in our study. Women working in the formal sector also experience this, but it is likely that women in informal employment with few social protections face greater hardship [15, 33]. Childcare, and breastfeeding more specifically, is generally thought to be an individual’s decision and success the sole responsibility of the woman. This ignores the role of society, including social and public services, in the support and protection of breastfeeding [3, 34]. Informal working mothers cannot and should not be expected to maintain exclusive breastfeeding without support. It is imperative that collective responsibility and positive attitudes towards breastfeeding be shared by caregivers, family members, and colleagues and co-workers within the informal economy. Communities where women live and work must recognize the importance of childcare in helping mothers manage complex situations. In addition, primary health care systems should also orient their services to working women and their needs rather than expecting women to respond to the configuration of the health system.

Evidence shows that women’s work and short maternity leave are leading factors contributing to women not establishing breastfeeding or early cessation of breastfeeding [3, 35, 36]. In many settings lack of support for continued breastfeeding in the workplace is associated with shorter durations of breastfeeding [6, 7, 35, 37, 38]. In our study, without any maternity benefits, and with strong pressure to earn for their households, mothers often returned to work soon after childbirth. In doing so mothers frequently reduced breastfeeding and opted to use breastmilk substitutes or to stop breastfeeding altogether. This supports the findings of Rollins et al. (2016), that working women in the informal economy are not appropriately supported through adequate maternity and workplace entitlements to be able to work and continue to breastfeed [3]. Further, mothers who took their child to work with them faced adverse environmental factors, as well as spatial and sociocultural challenges to breastfeeding precisely because of working in public. There were limited opportunities to breastfeed or express milk at the workplace and breastfeeding in public led to embarrassment and or was restricted in some cases. Mothers who brought their child to work risked loss of income because they might be distracted, or customers may have a prejudice against buying from a woman with a baby. Men in this study understood the importance of breastfeeding but conveyed an ambivalence and in many cases, antipathy towards breastfeeding in public. This has been found in a range of contexts [3].

Studies in formal employment settings suggest that the workplace is important in maintaining breastfeeding rates among working mothers and breastfeeding facilities in the workplace are associated with a higher probability of breastfeeding [39]. Characteristics of breastfeeding friendly workplaces include the availability of on-site nurseries, extended breaks, facilities to express and store milk, lactation rooms and lactation consultants or programmes [39]. Safe and supportive spaces in work and public spaces to enable closeness between a mother and her child are critical and can be customized to informal workplaces. This may include infrastructure and safe areas in public places, such as markets, as well as in private homes, in the case of domestic workers. These could range from play areas, lactation spaces, or places for a mother to breastfeed in private, rest and wash herself as needed. Working mothers in our study needed to have better options for breastfeeding, especially those working outside the home. This has been shown elsewhere in the Global South [35].

With respect to study limitations, identifying informal working mothers was a challenge. We used purposive and snowball sampling to address this constraint, however we acknowledge that this may have resulted in women with similar views being sampled. In addition, in South Africa we focused on market traders and domestic workers, so many informal work settings were not represented, including farm workers, construction workers and home-based workers.

## Conclusion

Our study shows that women informal workers face multiple challenges to breastfeeding their children leading to poor feeding practices that may adversely affect child health and development in this vulnerable population. Household income is frequently dependent on womens’ work and informal workers struggle to balance childcare needs with the need to work and provide for themselves and their family. Unless the challenges facing women working in the informal economy are addressed at the individual, household, community and municipal level, it is unlikely that global health and development goals, and global breastfeeding targets will be met. This will require removing the structural and societal barriers to child care and increasing the value that communities place on maternal and child health. Investments in social protection and public services are required, including child care, to support gender equality within families and society at large [34]. The next step of our multi-country collaboration is to address the interconnected pathways for intervention and support informal working mothers to sustain their livelihoods, protect their own health and nurture their children [17].

## Supplementary information

**Additional file 1:** Focus group discussion guide for female informal workers.

**Additional file 2:** Focus group discussion guide for male informal workers.

## Data Availability

The transcripts and study tools that support the findings of this study are available from the Centre for Rural Health and will be made available by the main and corresponding author on reasonable request.

## Abbreviations

AeT: Asiye eTafuleni
FGD: Focus group discussion
SEWA: Self-employed womens’ association
WHO: World Health Organization
