# Supplementary material for: Attitudes and perceptions about breastfeeding among female and male informal workers in India and South Africa
Source: BMC Public Health. 2020 Jun 5;20:875. doi: 10.1186/s12889-020-09013-9 (PMC7275335; doi:10.1186/s12889-020-09013-9)
Supplement: Supplementary file 1 — Additional file 1: Focus group discussion guide for female informal workers. [file 12889_2020_9013_MOESM1_ESM.pdf]

A study to explore child care and feeding practises among working women in the informal work sector

Focus group discussion guide: informal working women

> Thank you for agreeing to participate in this discussion. The reason for asking these questions is to hear the opinions of women who are working in an informal environment as well as having to care for young children. We want to understand your situation so we can develop appropriate solutions to support women who need to look after their children while working.
>
> So we are going to ask you some questions about your work, what work you do and the places where you do it. We are also going to ask about your children and how you care for them while you are working, and how you balance your work responsibilities with caring for your child. We will then ask you about what assistance and support could have been provided in the workplace to help you care for your child.
>
> We are particularly interested in young babies and the time after your baby was first born when you returned to work, so if your child is older, please think back to issues that affected you at that time

Introduction/background

1. **(Ice breaker) Please just tell everyone your child's name and something you especially love about your child.**

2. **Everyone here is employed in the informal sector and you all look after children too, so please can you tell us about the work you doing now and where you do it?**

   Prompt: Are you working at home or outside the home? Are you able to decide the hours that you work? Are you self-employed or do you have someone who employs you?

First we are going to explore what happened when you returned to work after your baby was born

3. **Can you tell the story about what happened to your baby when you went back to work after he/she was born?**
   Prompt: Did you stop working when the baby was born? For how long? What affected your decision about how long to be away from work after you gave birth to your baby? Are you able to take the child to work with you? Did you breastfeed the baby after he/she was first born? For how long?

4. **Describe how having a baby changed the work that you do?**
   Prompt: Did your work change? how much? and what kind of work did you do previously?

5. **Describe how going back to work changed the way you were feeding your baby?**

Prompt: how were you feeding before you came back to work? Did you change the way you were feeding the baby when you started working again? Are you able to breastfeed your baby while you are working? Why did you make this decision?

Now we are going to talk about how you experience working and looking after children

6. **Describe your experience of/what it is like being a working woman with a young child to care for?**
   Prompt: Does anyone help you to care for your child when you are working? Who oversees the care of the child on a day-to-day basis? Who is the carer? What is the place? Is it your home? Someone else's home? A crèche? Why did you chose this place? Are you happy with it?

7. **What do you think is important and needs consideration when you are deciding on how to feed your baby?**

   Prompt: what food should you give? Explore why participants made the choices that they did, either to formula feed or breastfeed. Why are decisions about feeding the baby important? How important do you think breastfeeding is? Explore how much do participants value breastfeeding and why?

8. **We are hoping to look for solutions for mothers in your situation to be able to continue to feed their babies with breastmilk once they have gone back to work. If you think about what happened when you started working, after your baby was born is there anything that could have helped you to breastfeed? Explain**

   Use a flipchart to write the solutions on the board. Use this to prompt discussions.
   Prompt: safe environment, privacy, hygiene etc.
