# Supplementary material for: Attitudes and perceptions about breastfeeding among female and male informal workers in India and South Africa
Source: BMC Public Health. 2020 Jun 5;20:875. doi: 10.1186/s12889-020-09013-9 (PMC7275335; doi:10.1186/s12889-020-09013-9)
Supplement: Supplementary file 2 — Additional file 2: Focus group discussion guide for male informal workers. [file 12889_2020_9013_MOESM2_ESM.pdf]

# A study to explore child care and feeding practises among working women in the informal work sector

## Focus group discussion guide for men

*We are trying to understand what men who work alongside women in the informal sector, or who are family members of working women, or (in the case of home-based workers) who are residents of the local community think about the need, importance and ability of working women to care for infants including and, in particular, breast-feeding.*

1. **What do you think is the role of women in the informal work environment?**

*Probe: is the role of women different from that of men? Do women have a role? Are there any areas where women should not work?*

2. **Tell me your views about women working within the informal work environment after they have had a baby?**

   *Probe: is this acceptable?*

3. **How should babies and young children be cared for while the mother is working?**

4. **How do you feel about babies being cared for in the workplace?**

   *Probe: How do you feel when you see mothers caring for babies in the work environment? How should mothers feed their babies while they are working? Describe the challenges that mothers may face in bringing a child to the workplace.*

5. **Describe any situation where you have seen a mother caring for a child in the workplace and how you feel about that?**

6. **How would you feel if you saw a mother breastfeeding her baby in the work environment?**

7. **What could be done to make it easier for informally working women to care properly for their infant while working?**
